# Stress-Softening in Particle-Filled Polyurethanes under Cyclic Compressive Loading

**DOI:** 10.3390/polym12071588

**Published:** 2020-07-17

**Authors:** Wenshuai Xu, Mangong Zhang, Yu Liu, Hao Zhang, Meng Chen, Heng Jiang, Yuren Wang

**Affiliations:** 1 Key Laboratory of Microgravity, Institute of Mechanics, Chinese Academy of Sciences, Beijing 100190, China; xuwenshuai@imech.ac.cn (W.X.); liuyu@imech.ac.cn (Y.L.); yurenwang@imech.ac.cn (Y.W.); 2 School of Engineering Science, University of Chinese Academy of Sciences, Beijing 100049, China; 3 Wuhan Second Ship Design and Research Institute, Wuhan 430064, China; zmg09@tsinghua.org.cn (M.Z.); zhyf1408@126.com (H.Z.)

**Keywords:** polyurethane elastomers, Mullins effect, spherical indentation, constitutive relationship

## Abstract

Elastomer compositions containing various particulate fillers can be formulated according to the specific functions required of them. Stress softening—which is also known as the Mullins effect—occurs during high loading and unloading paths in certain supramolecular elastomer materials. Previous experiments have revealed that the load–displacement response differs according to the filler used, demonstrating an unusual model of correspondence between the constitutive materials. Using a spherical indentation method and numerical simulation, we investigated the Mullins effect on polyurethane (PU) compositions subjected to cyclic uniaxial compressive load. The PU compositions comprised rigid particulate fillers (i.e., nano-silica and carbon black). The neo-Hooke model and the Ogden–Roxburgh Mullins model were used to describe the nonlinear deformation behavior of the soft materials. Based on finite element methods and parameter optimization, the load–displacement curves of various filled PUs were analyzed and fitted, enabling constitutive parameter prediction and inverse modeling. Hence, correspondence relationships between material components and constitutive parameters were established. Such relationships are instructive for the preparation of materials with specific properties. The method described herein is a more quantitative approach to the formulation of elastomer compositions comprising particulate fillers.

## 1. Introduction

Mechanical softening promotes energy dissipation and heat buildup in nanoparticle-reinforced elastomers. Such materials are used widely in engineering applications such as vibration reduction, heat preservation, damping improvement, and the development of composite materials [1,2,3]. Polyurethane (PU) elastomers—which are synthetic polymers with repeating units comprising urethane functional groups—play an essential role in industrial manufacture [4,5]. PUs are similar to natural rubber in terms of mechanical hysteresis, residual strain, and the stress-softening effect, and they have many excellent properties such as strong designability, good environmental adaptability, favorable damping characteristics, and biocompatibility [6,7,8,9,10,11]. When subjected to cyclic uniaxial loading, rubber-like materials exhibit an irreversible degradation in mechanical properties following the initial load, resulting in an obvious difference between the loading and unloading paths. Therefore, the stress required to achieve a certain elongation for the first time is always greater than the reloading stress. This phenomenon is an important indication of energy absorption; it has been widely studied and is known as the Mullins effect [12,13,14].

Many researchers have attempted to explain the Mullins effect and to establish models to describe it, such as the two-phase theory [15,16,17,18,19,20], the interface model [21,22,23], the superelastic damage model [24], and the pseudoelastic theory [25,26]. Particulate fillers such as nano-silica and carbon black (CB) can be used to reinforce rubber-like materials, as well as improve the energy absorption and heat dissipation of polymers. The Mullins effect has been investigated in nanoparticle-filled rubbery materials [17,27,28], but the use of nanoparticles as reinforcement agents for polymers remains controversial [29,30,31,32,33,34,35]. When nano-silica is added to a PU matrix, the hydroxyl groups on the surface of the nano-silica form a tightly cross-linked structure with the PU, which improves its adhesiveness, film hardness, tensile strength, and colloid viscosity [36]. By studying the mechanoluminescence of silica-filled elastomers subjected to cyclic loads, Clough et al. concluded that covalent bond scission contributes significantly to irreversible stress softening [37]. Shen et al. tested the Mullins effect in compressed PU foam and obtained a stress–strain curve that was consistent with the Ogden model [38]. Gavrilov et al. studied the mechanisms of filler reinforcement in elastomer nanocomposites; filler structure and filler breakdown were introduced to explain the nonlinear mechanical softening resulting from the loss factor and the presence of fillers [39]. Song and Zheng demonstrated the importance of microscopic interactions in the formation of the interface layer around the nanoparticles and the polymer bridges connecting nanoparticles [40,41]. They also found that the mechanism of nonlinear mechanical softening in filled rubber compounds and vulcanizates is rooted in the macromolecular chains of the entangled network rather than damage to the filler network or the filler–rubber interface. Using a combination of narrow- and wide-angle X-ray scattering methods during repeated in situ tensile loading, Sui et al. demonstrated that the Mullins effect in thermoplastic PUs is attributable to nano-scale fuzzy interfaces [36]. However, few studies have been carried out on the mechanical response of nanoparticle-filled polyurethanes (NPFPUs) subjected to compressive load conditions using the spherical indentation method. In particular, the influence of rigid nanoparticle fillers on the constitutive relationships in NPFPUs remains unclear.

The present study comprised an investigation of the mechanical responses of nanoparticle-filled and unfilled polyester polyurethanes to cyclic compressive load. The relevant characterization of the relationship between the Mullins effect and the volume of the filler fraction was ensured by using the spherical indentation method and the numerical inversion method. The indentation method is a simple approach to in situ local characterization at multiple levels, and it is a promising tool for research into the Mullins effect [42,43]; it can be used to investigate issues relating to linear elasticity, plasticity, hyperelasticity, and viscoelasticity [44,45]. A combination of theoretical analysis, finite element simulation, and experimentation has revealed the influence of various fillers on the Mullins effect, and the determining parameters of the neo-Hooke and Mullins models have been established.

The current paper is organized as follows. Section 2 describes the fabrication process and the material properties of the NPFPUs, defines the hyperelastic neo-Hooke model and the Ogden–Roxburgh Mullins model, and reports the loading conditions and measurements obtained during the mechanical tests. Section 3 compares and discusses the response results of the NPFPUs. A dimensional analysis of the relationships between the indentation responses and the properties of the materials was carried out, and the effective parameters were validated by numerical fitting. Section 4 provides a discussion of the results and the conclusions that can be drawn.

## 2. Materials and Methods

### 2.1. Preparation of the Filled Polyurethane Materials

The NPFPU materials were prepared by mechanical blending. To avoid uneven dispersion and ensure sufficient stirring, we chose a polyester prepolymer (component A) and a 4,4′-methylene bis(2-chloroaniline) (MOCA) curing agent (component B). The polyester prepolymer (Trademark H1342, viscosity 1100 mPa·s, density 1.25 × 10^3^ kg·m^−3^, Shore A hardness 90 at 25 °C, tear strength 100 N/mm, tensile strength 47 MPa, gelation time 15 min, melting point 80 °C) was purchased from Huatian Rubber & Plastic Technology Co., Ltd., Zibo, China. The MOCA curing agent (flaky solid, melting point 105 °C) was obtained from Chongshun New Material Technology Co., Ltd., Weifang, China. Hydrophobic fumed silica (CAS60676-86-0, specific surface area 1.15 × 10^5^ m^2^·kg^−1^, particle size 7–40 nm) and CB (N550) were obtained from Shanghai Macklin Biochemical Co., Ltd., Shanghai, China. Heat dissipation was accompanied by stirring and mixing, which speed up cross-linking reactions but are not conducive to filler dispersion. The ambient temperature during the sample manufacturing period was 25 °C. First, the solidified polyester prepolymer and MOCA were liquefied in an electric vacuum drying oven at 80 °C and 105 °C for 24 h, respectively. Then, the liquefied polyurethane prepolymer and nanoparticles were mixed at high speed (1000 rpm) for 10 min in a vacuum mechanical stirrer. The internal temperature of the vacuum agitator rose to 45 °C in this process. Then, molten MOCA (mass mixing ratio *A*:*B* = 100:12.4) was added to the homogeneous mixture, and vacuum stirring was continued for 5 min at 45 °C. During this process, air bubbles in the mixture were eliminated by multiple vacuuming operations. Finally, the cross-linked mixture was poured into a mold and placed in an electric vacuum drying oven at 80 °C for 12 h. This method is simple and is suitable for preparing hybrid materials and NPFPUs in various forms. We fabricated PU samples without any fillers, and with 5 parts per hundred resin (phr) and 10 phr nano-silica, and 5 phr and 10 phr CB; the samples were 72 mm in diameter and 60 mm in height. The density and shore A hardness of unfilled polyurethane sample are 1.105 × 10^3^ kg·m^−3^ and 92, respectively. The densities of NPFPUs with 5 phr nano-silica, 10 phr nano-silica, 5 phr CB, and 10 phr CB are 1.121 × 10^3^ kg·m^−3^, 1.130 × 10^3^ kg·m^−3^, 1.123 × 10^3^ kg·m^−3^, and 1.136 × 10^3^ kg·m^−3^; and the values of shore A hardness at 25 °C are 91.6, 94.6, 91.8, and 94.8, respectively.

### 2.2. Mathematical Model of the Mullins Effect

Before continuing, models describing hyperelasticity and the Mullins effect in unfilled polyurethanes (UFPUs) and NPFPUs are required. Many constitutive models—such as the neo-Hooke model, the Mooney–Rivlin model, the Yeoh model, the Gent model, and the Arruda–Boyce model—have been developed to simulate the hyperelasticity of soft matter [46,47,48,49]. Owing to the excellent mathematical fit they provide, the neo-Hooke and Ogden–Roxburgh Mullins models have been used to describe the Mullins effect in NPFPUs. No influence of chemical interactions or covalent linkages is considered for developing the mathematical model. It is essential to construct a suitable strain energy function, with variables including the stress invariant and the tensile ratio. In the neo-Hooke model, the strain energy density function *W* is indicated by

(1) $$W=C_{10}\left(I_{1}-3\right)+\frac{1}{D}{\left(J-1\right)}^{2},$$

(2) $$I_{1}={\bar{\lambda }}_{1}^{2}+{\bar{\lambda }}_{2}^{2}+{\bar{\lambda }}_{3}^{2},$$

where *W* represents the strain energy density per unit volume in the unstrained materials, *C*_10_ and *D* are material parameters, *J* is the volume ratio, and *I*_1_ is the first deviatoric strain invariant; and ${\bar{\lambda }}_{i}=J^{-\frac{1}{3}}\lambda_{i}$, where *λ_i_* and ${\bar{\lambda }}_{i}$ are the principal stretch and deviatoric principal stretch, respectively. The parameter *D* is related to the initial bulk modulus *K*_0_ by *K*_0_ = 2/*D*, with *D* = 0 for incompressible materials. The initial shear modulus *μ*_0_ is given by *μ*_0_ = 2*C*_10_. The Poisson’s ratio in the neo-Hooke model is *v* = (3*K*_0_ − 2*μ*_0_)/(6*K*_0_ + 2*μ*_0_).

The Ogden–Roxburgh model based on pseudoelastic theory has a small ratio of residuals to total deviation; it is superior in terms of fit, and it has been widely applied in commercial finite element software [26,27]. The essence of the model is that the elastic strain energy density function and various other strain energy functions are used to describe the loading process. The Mullins effect can be expressed by the following augmented energy function:

(3) $$U\left({\bar{\lambda }}_{i},\eta \right)=\eta U_{d}\left({\bar{\lambda }}_{i}\right)+\varphi \left(\eta \right)+U_{v}\left(J^{el}\right),$$

where *φ*(*η*) is a smooth continuous function of the damage variable referring to the damage function; the damage variable *η* changes with deformation, as expressed by

(4) $$\eta =1-\frac{1}{r}erf\left[\frac{1}{m}\left(U_{d}^{m}-U_{d}\right)\right],$$

where $U_{d}({\bar{\lambda }}_{i})$ is the deviatoric component of the primary hyperelastic strain energy density; *U_v_* (*J^el^*) is the volumetric component of the strain energy density; and *J^el^* represents the elastic volume ratio. $U_{d}^{m}$ is the maximum value of *U_d_* at the same load point during deformation. The parameters *r* and *m* can be assigned directly or matched by curve-fitting the cyclic compressive test results, and they are subject to the restrictions *r >* 1 and *m* ≥ 0 (*r* and *m* cannot both be zero), whereas *r* is dimensionless and *m* has the dimensions of energy; the error function *erf*(*x*) is defined as

(5) $$erf\left(x\right)=\frac{2}{\sqrt{\pi }}\int_{0}^{x}\exp\left(-\omega^{2}\right)d\omega .$$

### 2.3. Loading Conditions and Experimental Setup

**Figure 1:**
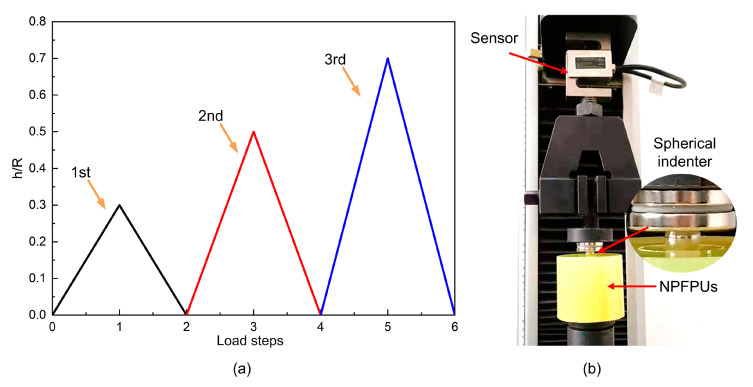
(**a**) Load steps in the cyclic compressive test; (**b**) Spherical indentation device.

The characteristic curves of the uniaxial cyclic compressive tests were obtained using a WDW-10E electronic universal test machine (Jinan Docer Testing Machine Co., Ltd., Jinan, China). The ambient temperature was 23 ℃ and the humidity was approximately 50% when the measurements were obtained. Spherical indentation, which has its origins in the hardness scratch test, uses spherical indenters to press into a material. The technique can be used to obtain information about a material’s mechanical properties, which can be used to produce load–depth curves. As shown in Figure 1b, the radius (*R*) of the spherical indenter was 5 mm. The loading and unloading conditions were continuously applied to produce indentations of various depths *h* (*h* = 0.3*R*, 0.5*R*, and 0.7*R*). The cyclic load programs were continuously set according to the same sequence: 0→0.3*R*→0→0.5*R*→0→0.7*R*→0 (Figure 1a); the loading rate was maintained at ±1 mm/min. Each specimen was tested three times at different points. A computer was connected to the experimental setup to record the data. The plasticity of the material was considered during base point calibration before each loading step.

## 3. Results and Discussion

This section describes the test results related to the Mullins effect and their analysis. The results were subjected to numerical fitting, and the key parameters were obtained from the constitutive equation. Hence, the functional data for the preparation and design of PU materials can be established before the commencement of the manufacturing process.

### 3.1. Experimental Results

**Figure 2:**
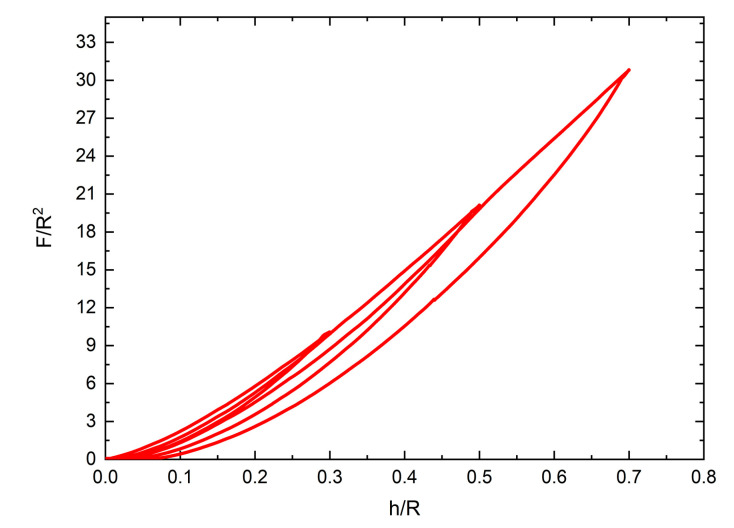
Test results for the unfilled polyurethanes (UFPUs) subjected to cyclic compressive loads.

**Figure 3:**
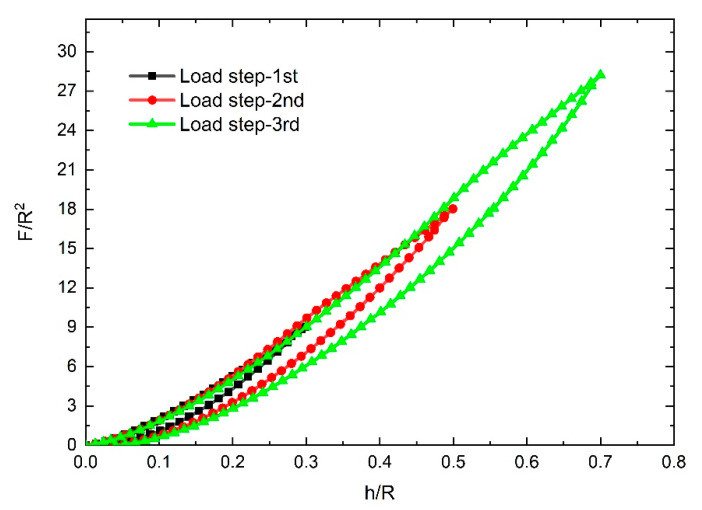
Corrected test results for the unfilled polyurethanes (UFPUs) subjected to cyclic compressive loads.

**Figure 4:**
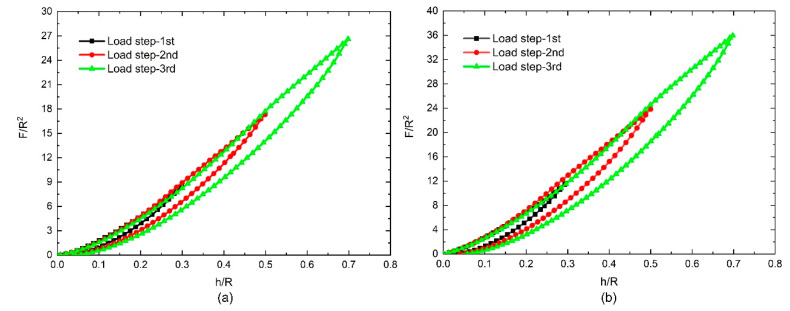
Test results for the nanoparticle-filled polyurethanes (NPFPUs) subjected to cyclic compressive load: (**a**) filled with 5 parts per hundred resin (phr) nano-silica; (**b**) filled with 10 phr nano-silica.

**Figure 5:**
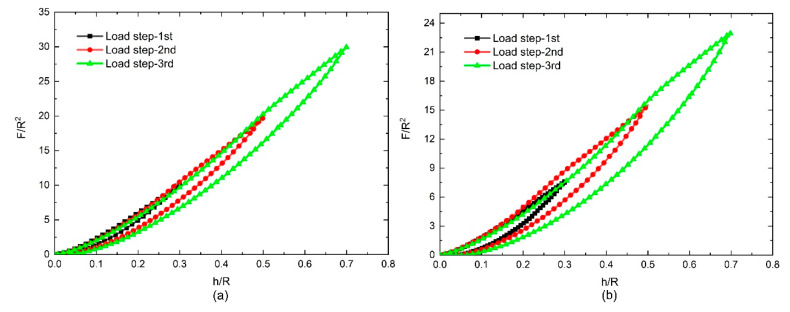
Test results for the nanoparticle-filled polyurethanes (NPFPUs) subjected to cyclic compressive load: (**a**) filled with 5 parts per hundred resin (phr) carbon black (CB); (**b**) filled with 10 phr CB.

The response curves of the NPFPUs subjected to cyclic uniaxial compressive loading are shown in Figure 2, Figure 3, Figure 4 and Figure 5. In contrast to the cyclic tensile test, the load–displacement curves were used to characterize the Mullins effect owing to the changes in the contact surface of the indenters during the loading process. During the “load–unload–reload cycle”, both the UFPUs and NPFPUs exhibited obvious Mullins effects. As clearly shown in Figure 2, the unloading and reloading stresses were much smaller than those during the initial loading step. During reloading, the load–displacement curve changed along the unloading path as the strain increased, as shown in Figure 2. Subsequently, as the strain increased further, the load–displacement curve coincided with the main curve. Apart from the Mullins effect, the residual plastic strain and permanent set (possibly due to viscoelasticity) occurred in both the UFPUs and NPFPUs, resulting in non-zero displacement after every loading cycle, and an initial no-load process before the next loading. The Mullins effect was the only hyperelastic issue considered in the present study. To avoid the initial no-load process, the experimental method was improved and modified to eliminate the influence of residual strain by recording test data from initial change of stress, rather than zero displacement. Therefore, the permanent deformation at the beginning of the load steps in Figure 2 should be ignored. Figure 3 shows the modified test data taking into account the residual plastic strain; the legends—e.g., “load step-1st”—indicate the numbers of the compressive load steps and the maximum displacement of the indenters corresponding to Figure 1. We performed dimensionless processing on the test data to facilitate the analysis described below, i.e., the load data (*F*) were divided by the square of the indenter radius (*R*^2^), and the indentation depth (*h*) was divided by the indenter radius (*R*).

**Figure 6:**
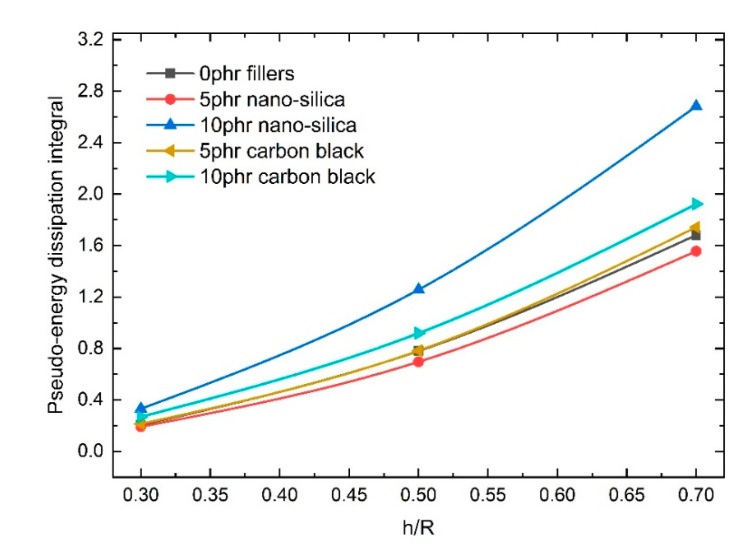
Integrals of the hysteresis loop areas from the compression tests.

The test results revealed that the load–displacement behavior observed during deformation typically included a significant strain energy contribution, and the primary loading–unloading cycle involved energy dissipation. We found that the loading curves of the three different steps overlapped into one loading curve, and hysteresis loops were formed between every loading and unloading step. As the load depth increased, the hysteresis loops became more obvious and changed according to the nanoparticle fillers used. Figure 4 shows the results for PUs filled with 5 phr and 10 phr nano-silica under cyclic compressive loads. The dimensionless load *F*/*R*^2^ changed according to the content of the filler; the dimensionless stress increased as the dimensionless displacement increased. At the same time, as the volume of the nano-silica filler increased, the dimensionless stress (*F*/*R*^2^) increased at the same displacement points and load steps. However, compared with the nano-silica-filled PUs, the CB-filled PUs exhibited more obvious effects under identical cyclic compressive loads. As shown in Figure 5, unlike in the nano-silica-filled PUs, the dimensionless stress (*F*/*R*^2^) in the PU filled with 10 phr CB was less than that in the PU filled with 5 phr CB. The largest residual strain appeared at the end of third load step in Figure 5b, which represents about 8% of the largest depth (*h*/*R* = 0.7). The others are much smaller than this ratio. We also found that the hysteresis loops—which can be regarded as pseudo-energy dissipation integrals—also varied according to the filler, as shown in Figure 6.

As shown in Figure 6, integration of the hysteresis loop areas revealed that the energy dissipation effect varied greatly among the NPFPUs. The dissipated energy indicated by the hysteresis loop area can be calculated using the following equation [38],

(6) $$\Delta E\left(n\right)=\int_{0}^{\varepsilon_{\max}}\left[\sigma_{load}\left(n,\varepsilon \right)-\sigma_{unload}\left(n,\varepsilon \right)\right]d\varepsilon ,$$

where ∆*E* is the dissipated energy of the hysteresis loop; *σ_load_* and *σ_unload_* are the loading stress–strain and unloading stress–strain functions, respectively; *n* is the number of cycles; and *ε* is the strain. In the present study, although the hysteresis loop derived from the dimensionless load and displacement differed from the strict stress–strain integral, it was used to characterize the energy dissipation capacity of the material. Hence, it can be regarded as a pseudo-energy dissipation integral. As the indenter displacement increased, the energy dissipation became more obvious. Specifically, compared with the UFPUs, the energy dissipation decreased in the 5 phr nano-silica-filled PU; when the filling ratio was increased to 10 phr, the energy dissipation was significantly greater than in both the 0 phr nano-silica- and 5 phr nano-silica-filled PUs. In the CB-filled PUs, the energy dissipation increased as the filler volume fraction increased. The Mullins effects in PUs with the same volume fractions of filler but with different fillers are distinct owing to unique filler networks; this law can be regular or irregular. Therefore, it is necessary to construct an equation describing the constitutive materials to determine the influence of nanoparticles. Such constitutive equations can be formulated through mathematical modeling and can be used to guide the functional design of materials.

### 3.2. Dimensional Analysis and Parameter Inversion

Dimensional analyses and finite element simulations establish explicit expressions for the relationships between indentation responses and material properties [50,51,52]. The dimensional analysis method is suitable for calculating dimensionless parameters with given variables when the equation is unknown, especially for situations in which only a few geometric and physical parameters are involved in the constitutive model. In the present study, the indentation load *F* is a function of independent parameters that can be expressed as *F* = *f* (*μ*_0_, *r*, *m*, *b*, *h*, *R*). Applying the П-theorem to *F* gives:

(7) $$F=\mu_{0}R^{2}\Pi \left(\frac{h}{R},r,\frac{m}{\mu_{0}},b\right)$$

It is difficult to solve the dimensionless function П by traditional analytical methods, but it can be determined by finite element simulations. Hence, different load–displacement curves can be obtained by changing the parameters *r*, *m*, *b*, and *C*_10_ in the neo-Hooke and Ogden–Roxburgh Mullins models. The optimized design software packages ISIGHT and SIMULIA Execution Engine can be used to design distributed calculations and determine the best design parameters [53]. Initial numerical parameters within the constraint value range given in Table 1 were assigned, and we obtained the final simulated parameters from the spherical indentation results.

The values in the constraint range of the practical parameters of concern varied widely; there was some regularity, but it was not immediately obvious. In the superelasticity neo-Hooke model, parameter *C* was larger in the UFPUs than in the PUs filled with 5 phr nano-silica, whereas the value of *C* increased in the PUs filled with 10 phr nano-silica. Parameter *C* was larger in the PUs filled with 5 phr CB than in the UFPUs and the PUs filled with 10 phr CB. Parameter *C* relates to the shear modulus of the material. One probable reason was that the framework of PUs with 10 phr nano-silica was more complete and stronger, causing a higher shear modulus. With the increase of CB mass fraction, poor dispersibility can cause particle aggregation and result in defects and uneven filler networks, which intensifies the softening of PUs. Parameter *r* was smaller in the nano-silica-filled PUs than in the UFPUs, and it was larger in the 10 phr nano-silica-filled PUs than in the 5 phr nano-silica-filled PUs. The value of *r* increased significantly when CB was used as the filler, and it was greater when 5 phr was used than when 10 phr was used. The particulate fillers and component proportions had a significant effect on parameter *m*. The change in parameter *b* was not obvious, which was a novel phenomenon. For the spherical nanoparticles used in the present study, the sizes were about dozens of nanometers. Therefore, the position and direction of particles are less correlated with the stress-softening phenomenon. In other words, the materials are isotropic [54]. In general, for the polyurethanes filled with nanoparticles, the interface between the particles and the polyurethanes molecules was important for the reinforcement effect, which was closely related to the type of filler and filling ratio.

**Figure 7:**
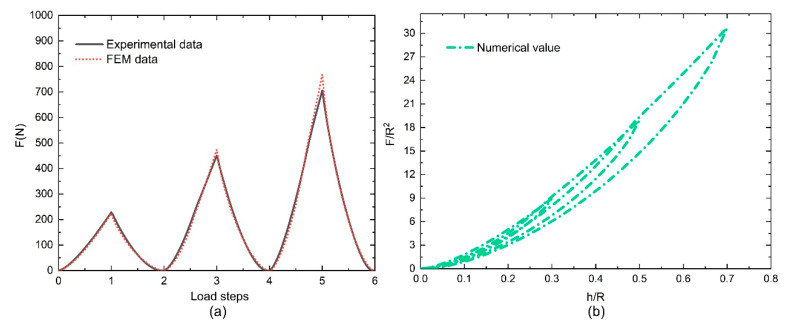
The numerical value curves for the unfilled polyurethanes (UFPUs) subjected to cyclic compressive load: (**a**) fitting the FEM data and the experimental data for the results of the loading steps; (**b**) numerical results of load-displacement curves. (FEM = finite element method).

**Figure 8:**
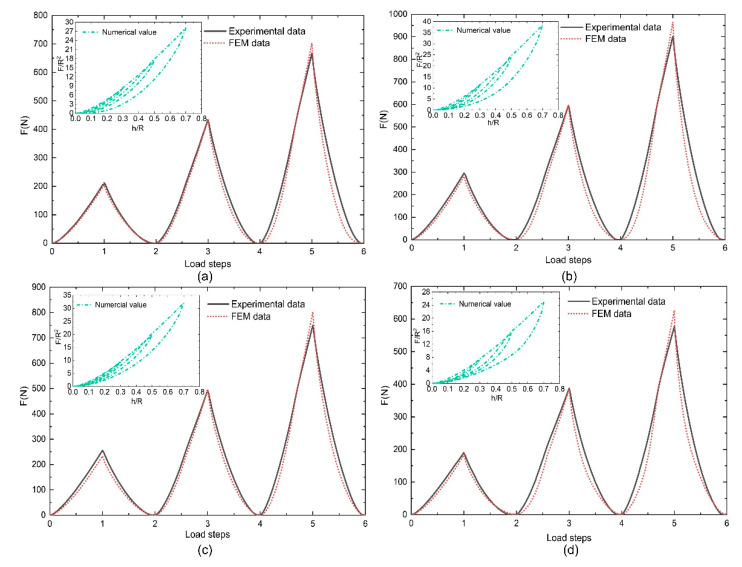
The numerical load steps and load–displacement curves for the nanoparticle-filled polyurethanes (NPFPUs): (**a**) filled with 5 phr nano-silica; (**b**) filled with 10 phr nano-silica; (**c**) filled with 5 phr carbon black (CB); and (**d**) filled with 10 phr CB.

In the results file, the historical variable output contains the load and indentation depth for the curve comparison with the target experimental data. The sum of the squares of the differences between the numerical loads and the target experimental load data is regarded as the basis for judging the results. The match between the final optimized load values and the actual measurements is shown in Figure 7a, and the final simulated load–displacement curve is shown in Figure 7b. Using the Hooke–Jeeves direct search method, the points near the current point were checked by disturbing the design variables continually to find the next improved point, with the objective of minimizing the sum of the squared differences; then, the procedure followed the most favorable direction until the calculator detected convergence. After the initial optimization step, the load–displacement curve of the simulated parameter was regarded as the target curve, and it was optimized with the experimental curves. Generally, the fitting parameters can be fully accepted when the mean square error between the two curves is less than 2%. The most suitable parameter value obtained after optimizing twice is given in Table 1; the final numerical load–displacement curves for the different fillers are shown in Figure 8.

As with the compression test results, the simulation results of the loading curves were basically independent of the loading strain, and their consistency was good. The unloading curve path depends on the maximum strain. The simulated load step curves coincided well with the experimental data, and they all basically overlap. However, the loading paths in the load–displacement curves did not completely coincide under the loading steps. Compared with the cyclic compressive curves in Figure 3, the numerical values matched better in the UFPUs. However, the other numerical results shown in the upper left corners of the curves in Figure 8 do not match very well. The coincidence in the unloading section is not as good as that in the loading section. Apart from algorithm accuracy, one possible reason for the unfavorable match is that the influence of the viscoelastic effect cannot be avoided completely in the test process. However, it was not considered in the numerical simulation. Results matching differs when the loading and unloading rates change, as already described and analyzed in Ref. [10].

## 4. Conclusions

In the present study, we used spherical indentation to investigate the Mullins effect and energy dissipation in PUs filled with different nanoparticles. The relationships between indentation loads, indentation depths, and the constitutive parameters of the materials used were established via theoretical, computational, and experimental methods. Based on the hyperelastic constitutive neo-Hooke and Ogden–Roxburgh Mullins models, we proposed the inversion constitutive parameters of the UFPUs and NPFPUs, and obtained a cyclic loading simulation curve. When subjected to a cyclic compressive load, the unloading and reloading stresses were much smaller than those during the initial loading, and the load–displacement curve coincided with the main curve as the strain increased. The proportion of particulate in a PU composition had a large but irregular influence on its properties, which means that it is not possible to judge the properties of NPFPUs according to the conventional viewpoint. Hence, using a combination of spherical indentation and numerical simulation, we established a material properties database that will provide visual theoretical references for function design. The spherical indentation method is also convenient for measuring irregularly shaped materials in real-life situations. The quantitative characterization of stress softening in the present paper will provide guidance for the design of high-performance nanoparticle-filled polymers, which will influence the properties of the final products and promote the development of polymer-damping devices.

## Figures and Tables

**Table 1:** Material parameters assignment conditions and final simulated parameters.

Model		Neo-Hooke	Ogden–Roxburgh Mullins		
Parameters		*C*_10_	*r*	*m*	*b*
Material parameters assignment condition	Constraint range	[0.5,10]	[1, 10]	[0.01, 5]	[0.01, 5]
	Initial of value	3	7	1	1
Final simulated parameters	0 phr fillers	5.0976	1.4314	0.08797	2.6609
	5 phr nano-silica	4.6416	1.0276	1.355	0.01
	10 phr nano-silica	6.3832	1.2734	0.97974	0.01
	5 phr CB	5.3052	1.8075	1.0138	0.01
	10 phr CB	4.1436	1.7826	0.28289	0.01
